# Atrial Septal Defect Device Closure With Balloon Support in a Patient With Situs Inversus With Dextrocardia

**DOI:** 10.7759/cureus.77156

**Published:** 2025-01-08

**Authors:** Aamir Rashid, Qayoom Yousuf, Syed Bilal, Imran Hafeez, Hilal Rather

**Affiliations:** 1 Cardiology, Sher-e-Kashmir Institute of Medical Sciences (SKIMS), Srinagar, IND

**Keywords:** atrial septal defect, balloon support, device closure, dextrocardia, situs inversus

## Abstract

This case report describes a rare and complex case of atrial septal defect (ASD) device closure in a 53-year-old female patient with situs inversus and dextrocardia, who presented with exertional dyspnea and palpitations. The procedure posed unique challenges due to the patient's mirror-image anatomy, requiring meticulous pre-procedural planning and advanced imaging. During the intervention, we used balloon support and modified fluoroscopic and echocardiographic views to account for the reversed orientation, ensuring precise device placement. This case highlights the importance of individualized approaches in managing structural heart defects in patients with complex congenital anomalies and underscores the feasibility of ASD device closure in such challenging anatomical conditions, providing insights for similar interventions in the future.

## Introduction

Situs inversus with dextrocardia, also known as situs inversus totalis, occurs in approximately one in 8,000 live births. Among patients with this condition who have concordant atrioventricular (AV) connections, the presence of an atrial septal defect (ASD) is very uncommon [1,2]. Conventional anatomical landmarks and orientations are reversed, necessitating careful planning and modification of standard procedural techniques. The left atrium is positioned more superiorly relative to the right atrium, with the atrial septum oriented in a more horizontal plane. The left pulmonary veins are much more anterior than expected [3]. We report a successful case of percutaneous ASD device closure in a patient with situs inversus and dextrocardia. Accurate diagnosis and procedural planning in such cases rely heavily on advanced imaging techniques. In this case, multimodal imaging, including transthoracic echocardiography (TTE) and transesophageal echocardiography (TEE), was crucial for assessing the anatomical orientation and size of the defect. Cardiac CT was also done, which detailed spatial visualization of the atrial septum and pulmonary veins anatomy, aiding in the selection and deployment of the device. Insights from previous literature guided the procedural planning, particularly in modifying the fluoroscopic views and echocardiographic imaging planes to account for the reversed anatomy.

## Case presentation

**Figure 1:**
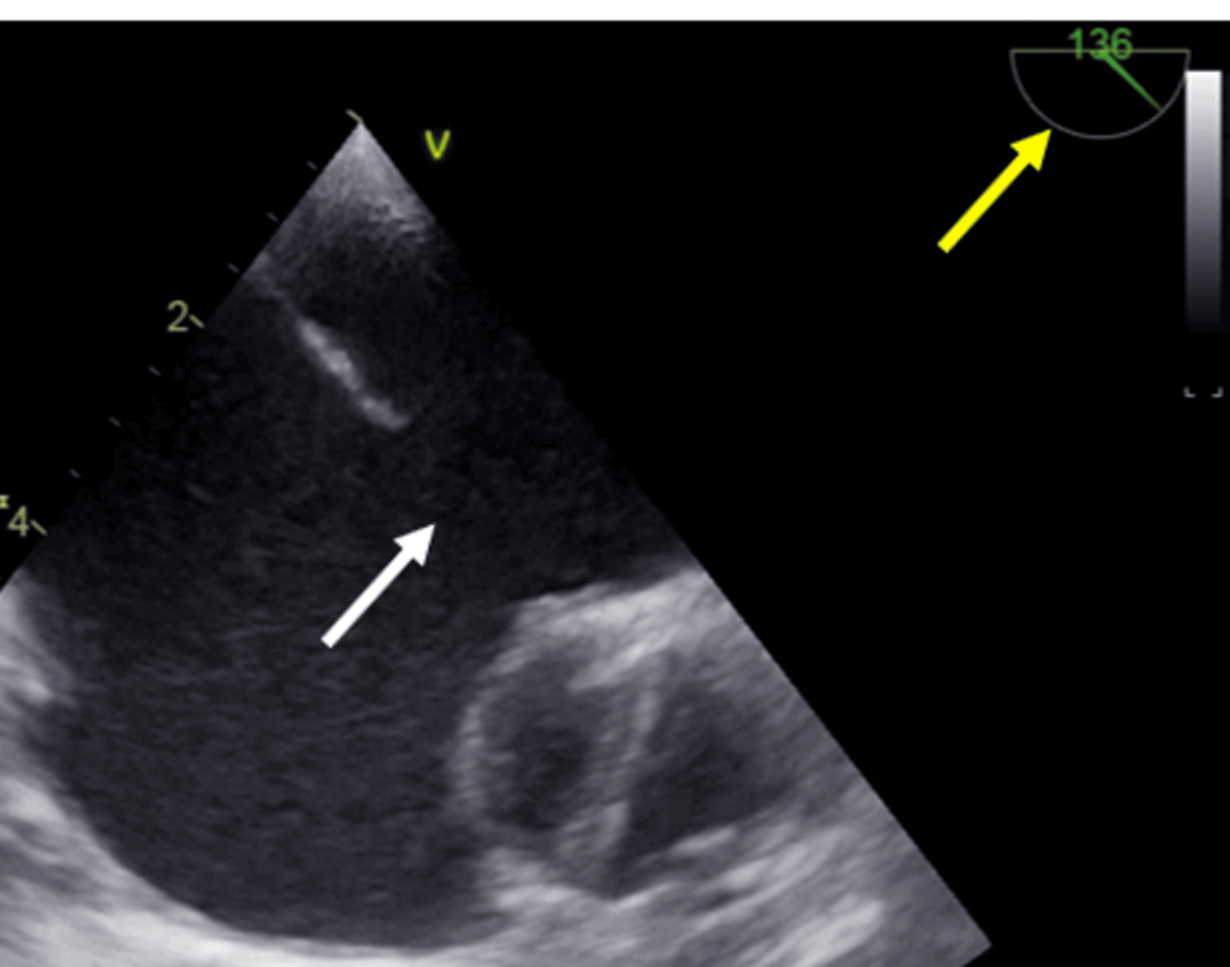
The transesophageal echocardiography (TEE) image obtained at a 130-degree angle mimics the typical appearance of a 60-degree angle due to the altered cardiac orientation. The white arrow indicates the large ASD, and the yellow arrow highlights the TEE imaging angle.

A 53-year-old hypertensive female, presented with a two-year history of progressively worsening dyspnea and exertional palpitations. The symptoms progressed from NYHA class I to NYHA class II over two years. There was no family history of congenital heart disease or other systemic conditions. Physical examination revealed a grade 2/6 systolic murmur along the right sternal border and a fixed split S2. Initially evaluated by a pulmonologist, she was diagnosed with suspected reactive airway disease and managed with bronchodilators. Persistent symptoms and cardiac signs prompted further imaging with chest X-ray and high-resolution computed tomography (HRCT), revealing situs inversus, dextrocardia, and cardiomegaly with dilated right atrium and ventricle. Electrocardiography showed normal sinus rhythm with incomplete right bundle branch block with abnormal p-wave axis and poor R wave progression in V1 to V6, which was suggestive of situs inversus with dextrocardia. The reversed precordial lead patterns reflect the anatomical mirror-image orientation. Echocardiography confirmed the situs inversus with dextrocardia with dilated RA and RV. There was a large 24 mm OS ASD with adequate rims. TEE images were reversed to properly profile the ASD (Figure 1).

**Figure 2:**
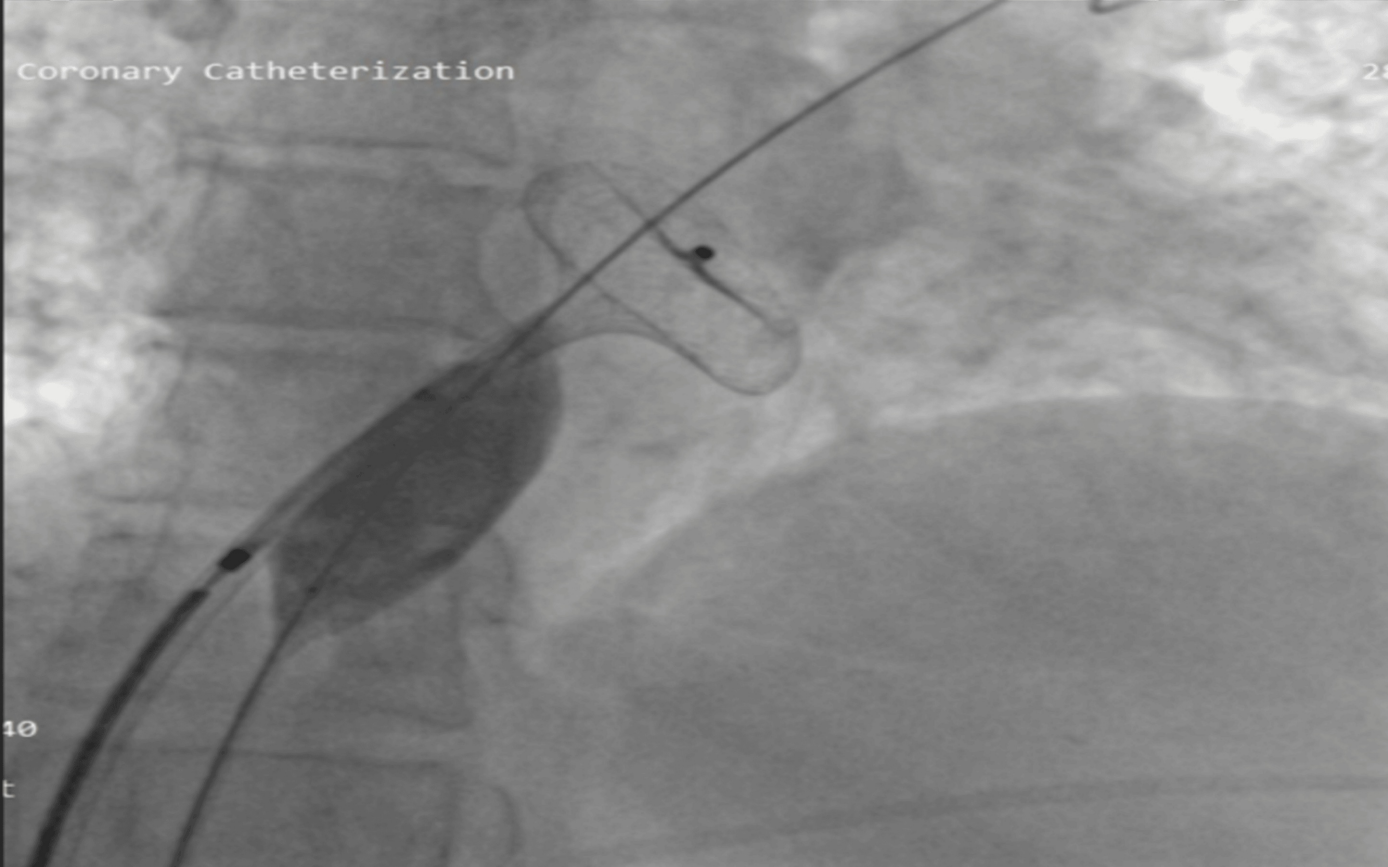
Anterior-posterior (AP) fluoroscopic view showing the atrial septal defect (ASD) device being deployed with balloon support.

The patient was planned for transcatheter ASD device closure. The projection process and procedural maneuvers were adapted as a reversed version of the standard approach, mirroring the typical technique. The mean pulmonary artery pressure was 35 mmHg. The ASD was crossed with a Judkin right (JR) catheter using Terumo (0.035) wire in LAO 30-degree angle with TEE guidance. Navigating the pulmonary vein during the procedure presented significant challenges due to its atypical orientation in the context of abnormal cardiac anatomy. Despite the difficulty, the JR catheter was successfully advanced and positioned within the right upper pulmonary vein. We also placed balloon support across the defect to prevent the device from slipping because of the complex anatomy. A 10 French ASD delivery was used to deploy a 28-size ASD occluder device using balloon support under TEE and fluoroscopic guidance (Figure 2).

The Minnesota maneuver was performed at the left anterior oblique 30 degrees, right anterior oblique 30 degrees, and in the TEE at 30° long axis, 90° bi-caval, 130° short-axis angulations with imaging guidance. TEE provided continuous imaging of the septum and defect, with fluoroscopy-guided catheter manipulation and device placement. Post-procedure TEE showed the device in situ with no residual shunt. Coordination between the two modalities ensured real-time feedback, enabling precise positioning and deployment of the device. Post-procedure, the patient was started on dual antiplatelets (aspirin (75 mg) plus clopidogrel (75 mg)) and advised to continue them for six months with regular echocardiographic follow-up at one month, six months, and one year to monitor device position and assess for residual shunting. She was counseled on maintaining a healthy lifestyle, including controlling hypertension, avoiding high-impact activities during the initial recovery period, and ensuring regular follow-ups for cardiac evaluation.

## Discussion

We report a successful case of percutaneous ASD device closure in a patient with situs inversus and dextrocardia using balloon support. Our case highlights the challenges and solutions in managing ASD in the unique anatomical context of situs inversus with dextrocardia. These conditions significantly alter conventional anatomical landmarks, making procedural navigation and device placement particularly challenging. Only a few cases have been reported of interventional closure of secundum ASD associated with situs inversus and dextrocardia [4-6]. This case contributes to current knowledge by demonstrating practical solutions to procedural difficulties posed by reversed anatomical landmarks, which are characteristic of situs inversus with dextrocardia.

While transcatheter ASD closure is a well-established procedure, its application in patients with complex anatomical variations, such as this case, remains underreported. This report fills a gap in the literature by highlighting specific modifications and strategies that can be applied to achieve successful outcomes. Successful transcatheter ASD closure in such a patient underscores the importance of meticulous pre-procedural planning, advanced imaging, and precise intraoperative management.

In patients with situs inversus and dextrocardia, the left atrium is positioned more superiorly, and the atrial septum is oriented horizontally, compared to its typical position [7]. The unusual orientation of pulmonary veins and cardiac chambers further complicates catheter navigation and device deployment. The reversed cardiac orientation necessitates modified procedural techniques and careful adaptation of standard equipment. The use of fluoroscopic and TEE guidance was pivotal in this case to overcome the challenges of reversed anatomy. Fluoroscopic angles were mirrored to adapt to the reversed cardiac orientation, and TEE images were reversed to accurately profile the atrial septum and guide device deployment.

The balloon support helps in controlling the disc movement and alignment of the device in anatomically complex cases. The inflated balloon helps in predictable alignment in the septum by allowing the left atrial disc to remain expanded over the left side of the septum while the right atrial disc fans out over the right side of the septum. This approach proved effective in this challenging anatomy and ensured optimal placement of the ASD occluder.

This case emphasizes that despite the anatomical complexity, percutaneous ASD device closure remains a viable and effective treatment option for patients with situs inversus and dextrocardia. We suggest the use of additional techniques like balloon-assisted technique and reversed imaging orientations for such complex cases. Procedural protocols emphasizing team-based approaches and customized imaging strategies can also provide a framework for addressing these challenges. It also highlights the importance of multidisciplinary teamwork, incorporating expertise in interventional cardiology, echocardiography, and imaging to achieve successful outcomes in such rare scenarios. While the procedure was successful, some aspects could be refined further. The use of advanced imaging techniques, such as 3D TEE or cardiac MRI, could have provided additional insights into the spatial relationships of cardiac structures. Real-time 3D visualization might also have reduced procedural time and enhanced precision.

## Conclusions

We report the successful transcatheter closure of a large ASD in a patient with situs inversus and dextrocardia, utilizing balloon assistance to facilitate the procedure. Post-procedure, there was the absence of residual shunt and significant symptomatic improvement. This case underscores the importance of meticulous pre-procedural planning and intraoperative management in addressing the unique anatomical challenges posed by situs inversus and dextrocardia. Demonstrating the feasibility and safety of this approach provides a valuable reference for clinicians encountering similar cases.

The use of balloon assistance proved pivotal in overcoming technical challenges and achieving optimal device placement, emphasizing its role as a viable adjunct in complex interventions. This adaptation demonstrates the potential for incorporating similar techniques into procedural guidelines and training programs for managing patients with situs inversus and dextrocardia. By addressing specific challenges like reversed anatomical landmarks and atypical pulmonary vein orientation, this approach offers a practical reference for clinicians handling comparable cases. Our case also highlights the value of team-based care, with interventionalists, echocardiographers, and imaging specialists contributing their expertise to overcome procedural challenges. This case contributes to the limited body of literature on ASD closure in this rare anatomical context, highlighting the potential for transcatheter techniques to be adapted successfully for challenging anatomies.
